# Detection of airborne viruses using electro-aerodynamic deposition and a field-effect transistor

**DOI:** 10.1038/srep17462

**Published:** 2015-12-08

**Authors:** Kyu-Tae Park, Dong-Guk Cho, Ji-Woon Park, Seunghun Hong, Jungho Hwang

**Affiliations:** 1 School of Mechanical Engineering, Yonsei University, Seoul 120-749, Korea; 2 Department of Physics and Astronomy, and Institute of Applied Physics, Seoul National University, Seoul 151-747, Korea

## Abstract

We report a technique for the detection of aerosolized viruses. Conventional field-effect-transistor (FET)-based techniques use solution-based processes, thus require antibody binding to the detection region of the FET prior to the supply of the analyte. With the method described here, virus–antibody-bound particles are delivered to the FET during detection; therefore, neither a pre-treatment antibody binding step on the FET channel nor washing process for virus–antibody-binding are necessary. Our method is based on the concept that virus–antibody-bound particles are larger than the virus or antibody alone, and thus have larger charge numbers following aerosol charging. When these particles are charged by negative ions and electro-aerodynamically deposited on a substrate, there exists a location on the substrate where neither lone virus nor antibody particles land, and where only virus–antibody-bound particles are deposited. If this location coincides with the channel of the FET, the resulting variation in the current can be used to indicate the existence of a virus. By aerosolizing a mixed solution of the virus and the antibody, only the virus–antibody-bound particles were transported to the swCNT-FET, and the electric current in the swCNT-FET decreased to 30% of that measured with no deposited particles.

Viruses are among the most important causes of human disease^1^,^2^,^3^,^4^ and present a growing concern as potential agents for biological warfare and terrorism^4^,^5^. Rapid, selective and sensitive detection of viruses is central to implementing an effective response to viral infections, such as through medication or quarantine. Established methods for viral analysis include plaque assays, immunological assays, transmission electron microscopy, and polymerase chain reaction (PCR) testing for viral nucleic acids^3^,^6^,^7^. These methods, however, cannot achieve rapid detection of a single virus; moreover, they often require a relatively high level of sample manipulation, which is inconvenient with infectious materials. Nevertheless, the ability to rapidly, directly and selectively detect individual virus particles would have a marked impact on healthcare by enabling diagnosis at the earliest stages of replication within a host system.

Exposure to biological aerosols (bioaerosols), such as those from H1N1 influenza, severe acute respiratory syndrome (SARS)^8^, bird flu^9^ and bioterrorism attacks^10^, has resulted in huge human and economic costs. Furthermore, the sustained growth in international travel increases the risk that an infectious disease may develop into a pandemic. These threats necessitate real-time bioaerosol sensing systems; however, development of such systems remains a challenge. Technologies including bioaerosol mass spectrometry (BAMS)^11^, surface-enhanced Raman spectroscopy (SERS)^12^ and flow cytometry with fluorochrome^13^ have been developed to detect bioaerosols. Fluorescence-based instruments, such as the ultraviolet aerodynamic particle sizer (UVAPS)^14^,^15^, BioTrak®^16^, and fluorescent microscopy with an inertial impactor^17^ can optically measure concentrations of total and/or viable particles in real-time. However, these techniques are not capable of species-level discrimination and/or produce high false-positive rates^18^. Surface plasmon resonance and Mie scattering with aerosol sampling have been also used for bioaerosol detection^19^,^20^. However, these methods need pre-treatments for binding antibody on a surface or particles.

A promising approach to the direct electrical detection of viruses is the use of field-effect transistors (FETs). Following recent advances in technology, the importance of high-performance electronic devices has increased. FETs are one of the most important components of current semiconductor technology, and have been applied in diverse fields outside of microelectronics. Such cross-disciplinary developments in technology provide an exciting opportunity for environmental sensing applications. FETs have been successfully applied to the detection of biological species in liquids via translating virus binding events into electrical signals. Changes in the conductance of the channel of a FET due to selective binding of specific proteins or nucleic acid sequences at the device surface have been reported using purified samples^21^,^22^,^23^. In addition, the considerable progress that has been made in microfluidic channels has enabled the efficient transport of virus-laden liquids onto specific-antibody-coated FETs^20^.

Current virus detection techniques that employ FETs are typically employing solution-based processes, and require the application of an antibody-binding process to the FET channel prior to the detection process (Fig. 1A). With such an antibody binding process, chemical treatment of the FET channel is carried out, followed by washing (Fig. 1A-a). A solution containing antibody particles for the target virus is then supplied to the FET channel (Fig. 1A-b). The reaction between the FET channel and antibody particles typically requires between 10 minutes and 3 hours. Following another washing step (Fig. 1A-c), the target virus-laden liquid (obtained by an aerosol sampling technique of capturing airborne viruses into a solution) is delivered to the FET to enable binding of virus particles onto the antibody particles (Fig. 1A-d). Then, another washing step is required to eliminate unbound virus particles (Fig. 1A-e). The presence of the bound virus particles changes the electrical characteristics of the FET, thereby implicating the existence of the virus (Fig. 1A-f).

Here we report a technique for the electrical detection of airborne virus particles without pre-treatment, without requiring antibody–antigen binding, and with no washing process involved in virus–antibody binding (Fig. 1B). Our method is based on the concept that both a virus and an antibody are particles of finite sizes. To realize our technique, a solution containing a target virus and another solution containing a specific antibody for this virus (Fig. 1B-a) must be prepared in advance, so that virus–antibody binding can occur (Fig. 1B-b). By atomizing the resulting mixture, the virus–antibody bound particles are aerosolized, as well as individual virus and antibody particles in the mixture solution (Fig. 1B-c). Water vapor and water droplets were removed by passing the aerosol flow through a diffusion dryer (Fig. 1B-d). The virus, antibody, and virus–antibody aerosols were electrically charged using a corona discharge (Fig. 1B-e), after which they pass through a nozzle and then toward the substrate (Fig. 1B-f). An external electric field is applied between the nozzle and substrate. With this electro-aerodynamic (EAD) deposition^24^, the motion of the particles is determined by inertial and electrical forces.

The smaller particles (i.e., virus and antibody) have relatively low charge numbers, whereas large particles (i.e., virus–antibody bound particles) have larger charge numbers^25^. Owing to the differences in size and charge, the smaller particles are deposited at locations nearer the flow-impinging zone (hereafter referred to as Zone 1), whereas the larger particles are deposited more widely across the substrate. Thus, there exists a location (hereafter referred to as Zone 2) where neither lone virus nor lone antibody particles land, rather where virus–antibody bound particles are deposited (Fig. 1B-g). The FET channel was located in Zone 2, and the change in source–drain current caused by the deposition of the virus–antibody bound particles was measured as an indicator of the presence of the virus (Fig. 1B-h). The effects of particle size on particle charge number and particle deposition width are mathematically explained in “Supplementary information”

## Results and Discussion

**Figure 2:**
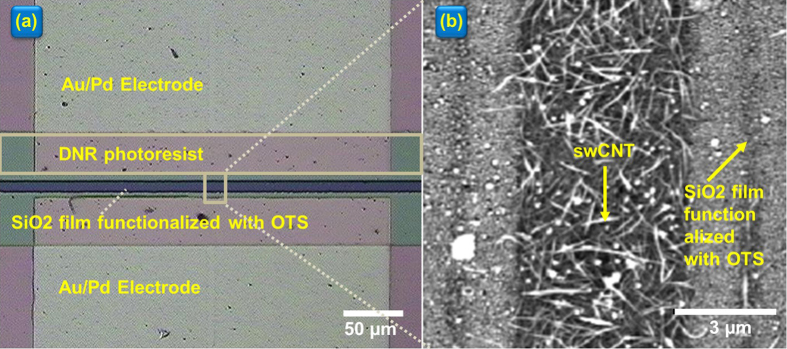
(**a**) Optical microscopy image of the swCNT-FET. (**b**) An AFM image of the swCNT channel region.

Figure 2(a) shows an optical image of a pristine single-walled carbon nanotube FET (swCNT-FET), which was obtained using optical microscopy (Sunny, Korea). Source and drain (Au/Pd electrodes) and SiO_2_ film functionalized with octadecyltrichlorosilane (OTS) are clearly evident in the image. Atomic force microscopy (AFM) was used to reveal the CNT structures in the channel region. Figure 2(b) clearly shows CNTs, which form a 5-μm-wide and 20-μm-long channel aligned between the source and drain electrodes.

**Figure 3:**
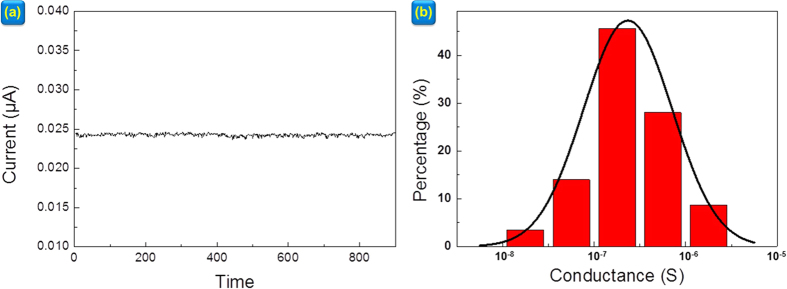
Stability and uniformity of swCNT-FETs; (a) Stability, (b) Uniformity.

Stability and uniformity tests of the swCNT-FET were carried out. For the stability test, the temporal variation of source-drain current in the swCNT-FET was measured for 15 minutes and found to be negligible (Fig. 3 (a)). For the uniformity test, seventy-six swCNT-FET samples were fabricated and the distribution in conductance was measured. Figure 3 (b) shows that the distribution followed a log-normal function, which is typical for percolating conductive networks^26^,^27^, and that the conductance was between 5 × 10^−8^ S and 1 × 10^−6^ S for 88% of the samples.

**Figure 4:**
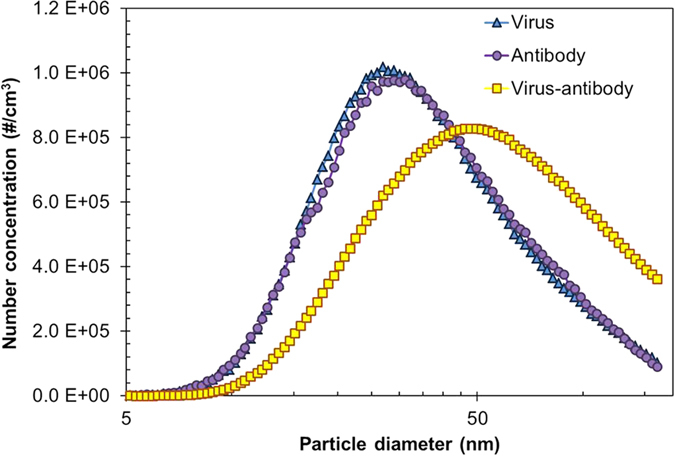
Size distributions of the virus, antibodies, and virus–antibody bound particles. The ratio of the number of virus particles to antibody particles was 1:1.

The particle size distribution was measured following aerosolization of a solution containing virus and antibody particles, and removal of moisture. Figure 4 shows three different size distributions obtained for solutions containing the MS2 bacteriophage virus, the antibodies for the MS2 bacteriophage, and mixture of both particles. The quantity of virus particles in the virus solution was controlled to be the same as that of the antibody particles in the antibody solution. The virus, antibody, and virus–antibody bound particles had modal diameters of 24 nm, 23 nm and 46 nm, respectively, and the total number concentrations were 6.7 × 10^5^ #/cm^3^, 6.72 × 10^5^ #/cm^3^, and 5.94 × 10^5^ #/cm^3^. Note that the virus and antibody particles were similar in size, and thus similar in terms of the total number concentration. When the mixed solution was aerosolized, the particle size distribution shifted toward larger particles, as expected; however, the total number concentration decreased compared with that obtained using the virus solution or antibody solution. This may be due to the fact that polyclonal antibodies were used, and so it is possible that unbound virus and antibody particles were present in the mixed solution.

After the particles were aerosolized, they were electrically charged using a corona charger. The charged particles entered the nozzle, and were accelerated at the exit with a velocity of 1.17 m/s, and impinged on the substrate. We have previously shown^24^ that the deposition domain on the substrate is circular, and that the diameter (or width) of the deposition domain is given by:


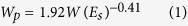


where *W* is the width of the nozzle width and *E*_*s*_ is the electrostatic number; i.e.,


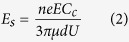


where *e* = 1.6 × 10^−19^ C is the elementary charge, *E* is the electric field strength, μ is the viscosity of air, *U* is the air velocity at the nozzle, *d* is the particle diameter, and *C*_*C*_ is the Cunningham slip correction factor; i.e.^25^,


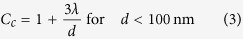


where *λ* is the mean free path of air molecules (66 nm). The average charge number of particles, *n*, was determined both theoretically and experimentally; details of this can be found in the ‘Methods’ section.

The average charge numbers were calculated using Eq. (5) (see the ‘Methods’ section); these were 3.31 for the virus, 3.16 for the antibody and 6.63 for the virus–antibody bound particles. These numbers are in good agreement with those obtained experimentally using Eq. (4); i.e., 3.05, 2.97, and 6.79, respectively. Therefore, we found that the virus and antibody particles had a similar charge number in addition to their similar size.

**Figure 5:**
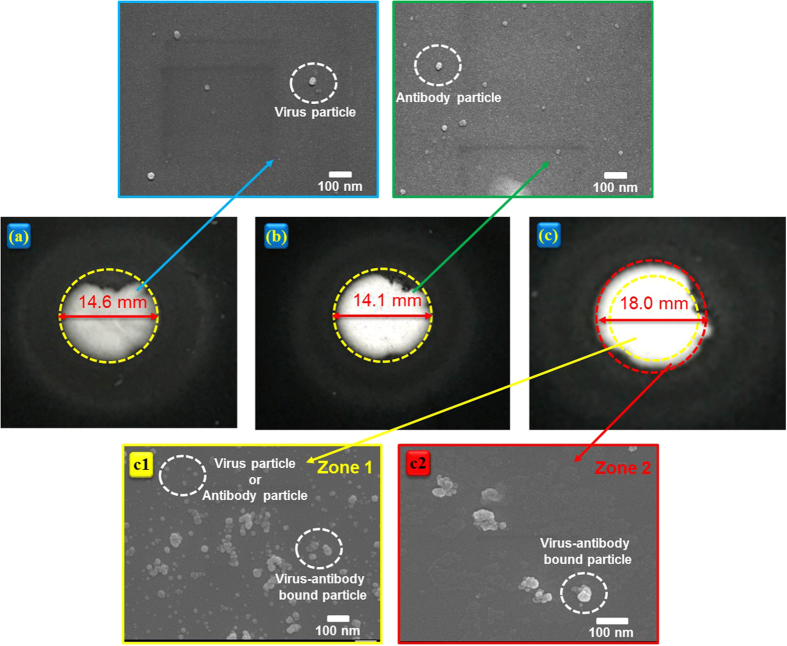
Photograph and SEM images of deposition spots using (**a**) a solution containing only the virus, (**b**) a solution containing only antibodies, and (**c**) a mixed solution containing both virus and antibodies.

The deposition widths for the aerosolized particles were calculated using Eq. (1); we find *W*_*p*_ = 14.1 mm for the virus, *W*_*p*_ = 13.9 mm for the antibodies and *W*_*p*_ = 17.4 mm for the virus–antibody bound particles. The deposition widths were measured using a digital camera (D80, Nikon, Japan). A black enclosure was installed near the deposition location to shield the region form external light. The measured deposition widths were 14.6 mm for the virus, 14.1 mm for the antibodies and 18 mm for the virus–antibody bound particles. Therefore, the measured data were in good agreement with the results of the calculations. Figure 5 also shows scanning electron microscope (SEM) images for four deposition spots. As shown in Fig. 5(a,b), the virus and antibodies were deposited as single particles. Virus, antibodies and virus–antibody bound particles co-existed in Zone 1 (*W*_*p*_ < 14.6 mm; see Fig. 5(c1)); however, only virus–antibody bound particles existed in Zone 2 (14.6 mm ≤ *W*_*p*_ ≤ 18 mm; see Fig. 5(c2)). Therefore, the swCNT-FET channel was located in Zone 2 in subsequent experiments.

**Figure 6:**
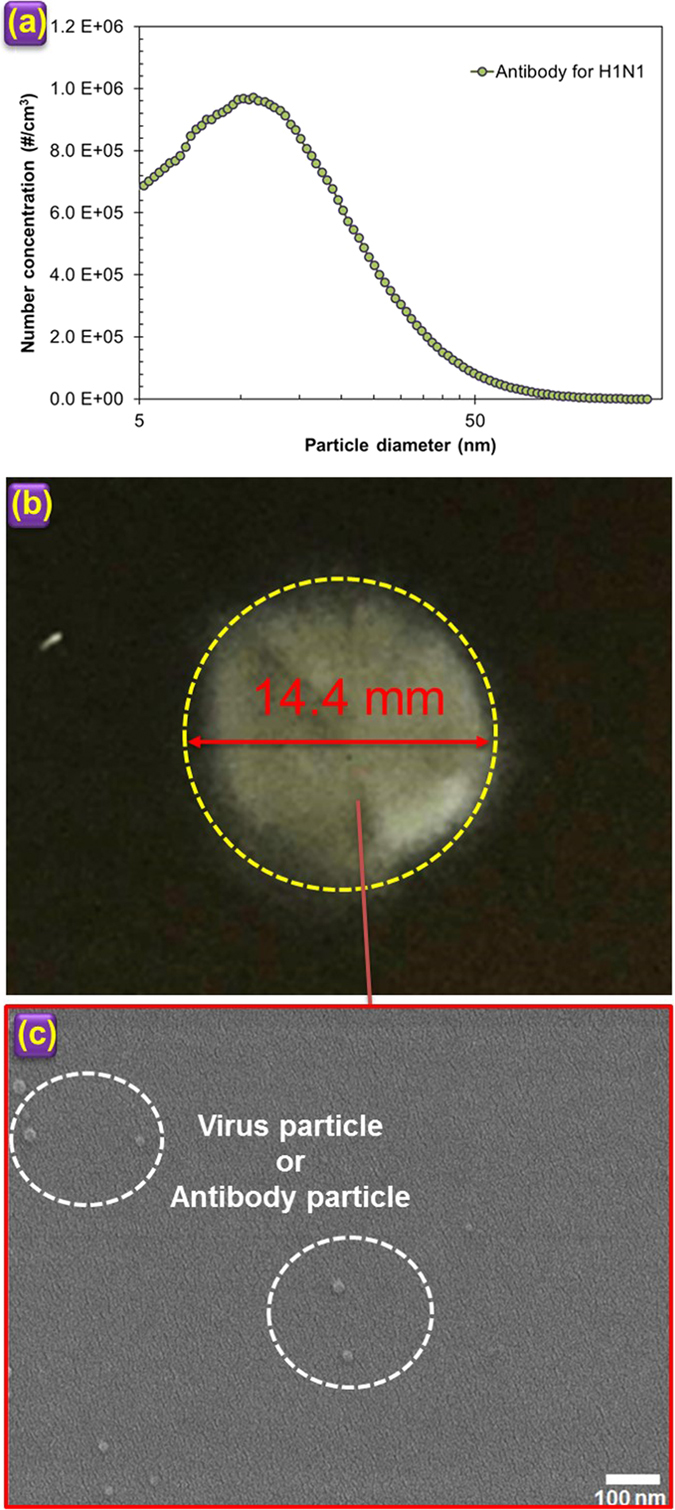
(**a**) The size distribution of the H1N1 antibodies. (**b**) A photograph of deposition location. (**c**) An SEM image of the deposited particles.

Additional experiments were carried out using antibodies specific for the H1N1 virus, and not for the target bacteriophage MS2 virus. Figure 6(a) shows the size distribution of the aerosolized antibodies for the H1N1 virus. The particles had a modal diameter of 11 nm and a total number concentration of 6.58 × 10^5^ #/cm^3^. A solution containing the target bacteriophage MS2 virus and antibodies for the H1N1 virus was prepared and aerosolized, and the aerosolized particles were electro-aerodynamically deposited. The deposition width was 14.4 mm (Fig. 6(b)), which is almost identical to that for the virus deposition shown in Fig. 5(a). Figure 6(c) shows an SEM image of deposition; single particles can be seen deposited on the substrate, and particle agglomeration was not observed. It follows that no change in the size of the particles due to binding between the target bacteriophage MS2 virus and the antibodies for H1N1 occurred.

**Figure 7:**
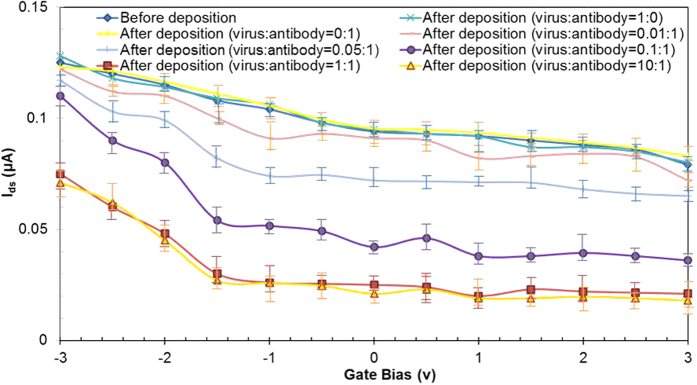
I-V characteristics of the swCNT-FET before and after the deposition of MS2 bacteriophage virus–MS2 bacteriophage antibody bound particles.

Figure 7 shows the source–drain current as a function of the gate voltage (i.e., *I-V* characteristics of the swCNT-FET) before and after the deposition of the virus–antibody bound particles. Figure 7 shows a dramatic decrease in the current following deposition of virus–antibody bound particles. When the number concentration ratio of virus to antibody was 1:0 or 0:1, the current did not change from that measured with no particle deposition (control value). However, when the ratios were 0.01:1, 0.05:1, and 0.1:1, the current decreased to 93%, 87%, and 46% of the control value, respectively, supporting that only virus-antibody bound particle can affect the current change. When the ratio further increased to 1:1 or 10:1, the current decreased to 30% of the control value on account of the higher number concentration of virus particles.

**Figure 8:**
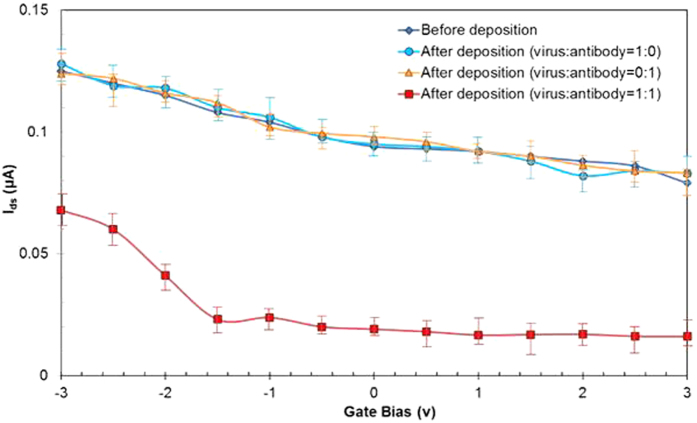
I-V characteristics of the swCNT-FET before and after the deposition of H1N1 virus–H1N1 antibody bound particles.

Detection of H1N1 virus was carried out to check the universality of our proposed method. The H1N1 virus, antibody for H1N1, and virus–antibody bound particles had modal diameters of 21.5 nm, 11 nm, and 31 nm, respectively, and the total number concentrations were 6.81 × 10^5^ #/cm^3^, 6.58 × 10^5^ #/cm^3^, and 5.83 × 10^5^ #/cm^3^. The charge numbers for H1N1 virus, antibody for H1N1, and virus-antibody bound particles were 2.85, 1.44, and 4.31, respectively. Since the calculated deposition widths were 13.3 mm, 10.7 mm and 15.7 mm, respectively, for H1N1 virus, antibody for H1N1 virus, and virus-antibody bound particles, the swCNT-FET channel was located in the domain between 10.7 mm and 15.7 mm. Then particle deposition experiments were carried out and the change in source–drain current was observed. Figure 8 shows 20% decrease in the current after the deposition of the virus–antibody bound particles.

The reduced current in the swCNT-FET that was observed in the presence of virus–antibody bound particle was most likely due to donation of electrons from negatively charged virus–antibody bound particles (see Fig. S2 in “Supplementary information”). Because of the *p*-type characteristics of the swCNT, donation of electrons would reduce the number of holes (*p*-type charge carriers) in the swCNT, resulting in a decrease in the source–drain current^28^.

## Conclusion

We have fabricated swCNT-FETs with an OTS SAM coating, and demonstrated specific detection of aerosolized bacteriophage MS2 using anti-enterobacteriophage MS2 coat protein antibodies. Following deposition of the (negatively charged) virus–antibody bound particles on the *p*-type swCNT-FET channel, the source–drain current decreased to 30% of that measured with no particles deposition, which enabled detection of the presence of the target virus. Moreover, for the test of universality of our detection method, H1N1 virus was also detected and showed the source–drain current decreased to 20% of that measured with no particles deposition.

## Methods

### Fabrication of swCNT-FET

We fabricated FETs using *p*-type single-walled CNTs swCNTs. A suspension of swCNTs was prepared by dispersing 5 mg of swCNTs (Hanwha, Korea) in 50 ml of 1,2-dichlorobenzene. To pattern the swCNT channel on a silicon dioxide wafer, an OTS self-assembled monolayer (SAM) technique was used. AZ5214 photoresist was patterned on the silicon oxide wafer using conventional photolithographic techniques. The wafer was then placed in a solution of OTS (1:500, v/v in hexane) for 5 min to form the OTS SAM on the wafer. The OTS-coated wafer was then dipped in acetone to remove the AZ5214 patterns, which resulted in an OTS-patterned wafer. The OTS-patterned wafer was then placed in the swCNT solution for 30 s, and rinsed using 1,2-dichlorobenzene. The swCNTs were then selectively absorbed onto the bare silicon dioxide regions, whereas the OTS SAM regions blocked the absorption of the swCNTs. Source and drain electrodes (Au/Pd, 30 nm/10 nm) were fabricated using conventional photolithography, thermal evaporation and lift-off. The source and drain electrodes were passivated using photoresist. Further details of swCNT-FETs can be found in Lee *et al*.^29^.

### Preparation of the test virus, antibodies and mixed solutions

Bacteriophage MS2 (ATCC 15597-B1) and anti-enterobacteriophage MS2 coat protein antibody were used as the target virus and specific antibody. Bacteriophage MS2 is commonly used in aerosol experiments as a surrogate for human and animal viruses^30^. Bacteriophage MS2 is nonpathogenic, can be prepared in high concentrations, is ideal for detection in aerosol experiments, and responds to antimicrobial agents in a manner that is similar to human viruses^31^.

*Escherichia coli* strain C3000 (ATTCC 15597) was selected as the host bacterium. To recover bacterial cells from a freeze-dried state, 10 ml of tryptic soy broth (TSB) were mixed with the freeze-dried bacterial cells. The mixture was incubated for 24 h at 37 °C with agitation. A total of 0.1 ml of the incubated bacterial solution was injected into another 10 ml of TSB. The TSB solution containing the bacteria was then used as the host bacterial solution following incubation for 6 h at 37 °C with agitation.

TSB (1 ml) was injected into the freeze-dried MS2 virus, and 0.1 ml of the viral solution was extracted. The extracted solution was mixed with 0.3 ml of the host bacterial solution and 29 ml of soft tryptic soy agar (TSA), containing 8 g/l agar. The resulting agar solution was poured into a Petri dish and incubated overnight at 37 °C. The surface of the agar was removed using 10 ml of phosphate-buffered saline (PBS) at pH 7.0. The solution was centrifuged for 20 min at 5,000 *g*, and the supernatant was used as the virus solution in subsequent experiments. The virus solution was diluted using PBS to control the number concentration of the virus particles. Virus solutions with virus: antibody number concentrations of 0.01, 0.05, 0.1:1, 1:1, and 10:1 were prepared. Influenza A (H1N1) virus, strain A /Beijing/262/95 (8IN73-2, HyTest, Finland) was prepared for universality test. H1N1 virus solution was prepared with 10 ml of PBS and 120 μl of H1N1 virus stock,

Anti-enterobacteriophage MS2 coat protein antibodies (ABE76, Millipore, Germany) and monoclonal mouse anti-influenza virus type A (3IN5, HyTest, Finland) were used. The MS2 coat protein of bacteriophage is an RNA-binding protein that assembles into a pentameric structure to form the phage shell. The MS2 coat protein binds to a stem-loop structure in the viral RNA and facilitates packaging of RNA molecules into viral capsids. In addition, it represses synthesis of the viral replicase enzyme. The MS2 coat protein is widely used as an experimental model for the study of RNA–protein interactions that are involved in virus assembly. With the H1N1 antibodies, hybridoma clones were derived from hybridization of Sp2/0 myeloma cells with spleen cells form BALB/c mice immunized with purified influenza virus type A strain H1N1. Two antibody solutions were prepared: one from 10 ml of PBS and 120 μl of anti-enterobacteriophage MS2 coat protein stock, and the other from 2.5 ml of PBS and 195 μl of H1N1 antibody stock. The virus and antibody solutions were then mixed and incubated at room temperature for 10 min.

### Experimental setup

Details of the experimental setup are shown in Fig. S3 of the ‘Supplementary Information.’ After the mixed solution was prepared, the particles were aerosolized using the following procedure. Compressed air was passed through a clean air supply consisting of an oil trap, a diffusion dryer, and a high-efficiency particulate air (HEPA) filter. The particle-free compressed air then entered a Collison-type atomizer (9302, TSI Inc., USA), containing the mixture. The aerosol flow rate was controlled using a rotameter. Dry, clean air flowed at 2 lpm, forming a high-velocity jet through an orifice in the atomizer. The pressure drop from this jet was used to draw the mixed solution up through a tube, which was then broken into droplets due to the high-velocity air jet. The resulting larger droplets impinged on an impactor, whereas the smaller droplets made no contact and formed an aerosol that exited through an outlet. The aerosolized mixed solution was passed through a diffusion dryer to remove water and a neutralizer (Soft X-ray charger 4530, HCT, Korea) to induce a Boltzmann charge distribution.

The neutralized particles entered a unipolar aerosol charger, with a stainless-steel needle (wire) electrode located at the center, which was used to generate a corona discharge at the sharp tip. The corona discharge generated air ions, which moved along the electric field to a grounded cylinder (made of duralumin). The charge per particle was controlled using a direct current (DC) voltage supplied from a high-voltage power supply. A bias of −8 kV was applied to the needle to charge the particles. Details of the charger are described in Park *et al*.^32^.

The charged particles (i.e., virus, antibody, virus-antibody) then entered a 30-cm-long cylindrical nozzle (6 mm in diameter), which was used to induce a fully developed flow to minimize the entrance effect at the nozzle (which had a Reynolds number of Re = 700). The nozzle filet had a radius of curvature of 2 mm at the tip to minimize the electric field^33^. After the charged particles were ejected from the nozzle, they were guided by an electric field and deposited on the substrate, which was located on an electrode that was biased at 7 kV. The separation between the nozzle and the substrate was 10 mm. The swCNT-FET was located where only the virus–antibody bound particles were deposited (i.e., in Zone 2).

The virus–antibody bound particles were deposited on the channel of the swCNT-FET, which led to a decrease in the source–drain current in the swCNT-FET. To measure the current, the swCNT-FET was connected to a semiconductor parameter analyzer (4200, Keithley, USA). A back-gated biasing method was used; the gate bias was varied in the range −3 V to + 3 V, and a constant source–drain bias of 100 mV was applied.

Aerosol sampling was carried out upstream of the nozzle. A scanning mobility particle sizer (SMPS) system was used to characterize the size distribution of the particles, which consisted of a differential mobility analyzer (DMA, 3081, TSI, USA) and a condensation particle counter (CPC, 3025, TSI, USA), which could measure the diameter in the range 4–165 nm. The SMPS system was operated with a sample flow rate of 0.3 lpm and a scan time of 180 s.

### Average particle charge

The average particle charge was characterized by measuring the electric current resulting from the charged aerosol using an aerosol electrometer (3068A, TSI, USA), which consisted of a Faraday cup and an electrometer. Particles in the sample flow were collected in a high-efficiency conductive filter housed in a metal enclosure, which constituted the Faraday cup. Charged aerosols entered the instrument through an outer metal housing, which shielded the electrometer input from external electric fields. An absolute filter was used to remove the charged particles from the air stream, which was enclosed in an inner metal housing and electrically insulated from the outer metal housing. A signal feed connected the filter to the input of a solid-state electrometer amplifier. The electrometer had an analog output voltage range of 0–10 V, which corresponded to a maximum current of −12.5 to +12.5 pA. An ion trap was used to remove free unattached ions, and to enable charged particles to pass through the ion trap zone, where the ion trap voltage was 200 V^34^.

The average charge number of charged particles was calculated as follows:


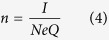


where *I* is the measured current, *Q* is the flow rate, and *N* is the total number concentration of charged particles.

The average charge number was also calculated using the following expression^24^:


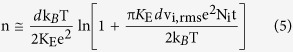


where *v*_*i*,rms_ = 240 m/s is the mean thermal speed of gaseous air ions, *K*_*E*_ = 9.010^9^ Nm^2^/C^2^ is the electrostatic constant of proportionality, *k*_*B*_ is Boltzmann’s constant, *d* is the mean particle diameter, *T* is temperature, and *t* = 0.0133 s is the particle residence time. The concentration of corona-generated air ions was determined as follows:


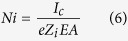


where *I*_*C*_ = 1.21 × 10^−7^ A is the corona current, *Z*_*i*_ = 1.6 cm^2^/Vs is the mobility of the gaseous ions, and *A* = 9.84 × 10^−7^ m^2^ is the internal surface area of the ground electrode.

## Additional Information

**How to cite this article**: Park, K.-T. *et al*. Detection of airborne viruses using electro-aerodynamic deposition and a field-effect transistor. *Sci. Rep*. **5**, 17462; doi: 10.1038/srep17462 (2015).

## Supplementary Material

Supplementary Information

## Figures and Tables

**Figure 1:**
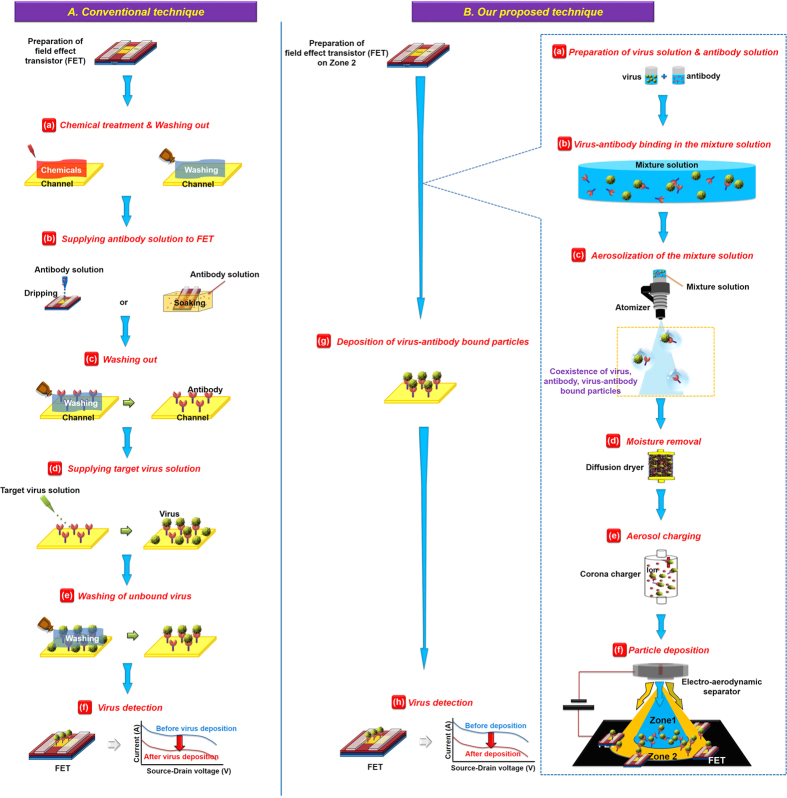
Electrical detection of the target virus using a specific antibody (K. –T. Park drew the figure by using Microsoft PowerPoint).
