# Supplementary material for: Detection of airborne viruses using electro-aerodynamic deposition and a field-effect transistor
Source: Sci Rep. 2015 Dec 8;5:17462. doi: 10.1038/srep17462 (PMC4672335; doi:10.1038/srep17462)
Supplement: Supplementary Information [file srep17462-s1.pdf]

Supplementary Information

Detection of airborne viruses using electro-aerodynamic deposition and a field-effect transistor

*Kyu-Tae Park[1], Dong-Guk Cho[2], Ji-Woon Park[1], Seunghun Hong[2] and Jungho Hwang[1]**

[1]School of Mechanical Engineering, Yonsei University, Seoul 120-749, Korea

[2]Department of Physics and Astronomy, and Institute of Applied Physics, Seoul National University, Seoul 151-747, Korea

KEYWORDS: aerosol, electro-aerodynamic deposition, single-walled carbon nanotube field-effect transistor (swCNT-FET), virus detection

Effects of particle size on particle charge number and particle deposition width

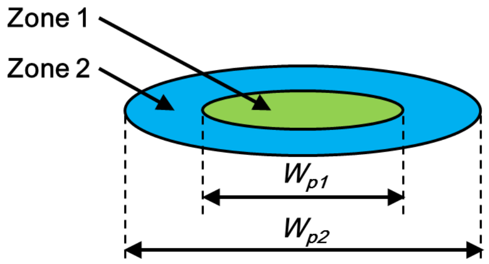

Figure S1. Deposition area of two different size particles

Using Eq.(1) and Eq.(2), the ratio between W_{p1} and W_{p2} is expressed as follows,

$$\frac{W_{p1}}{W_{p2}} = \left(\frac{\frac{3\pi\mu d_1 u}{n_1 E C_{C1}}}{\frac{3\pi\mu d_2 u}{n_2 E C_{C2}}}\right)^{0.41} = \left(\frac{\frac{d_1}{n_1 C_{C1}}}{\frac{d_2}{n_2 C_{C2}}}\right)^{0.41} = \left(\frac{d_1 n_2 C_{C2}}{d_2 n_1 C_{C1}}\right)^{0.41} = \left(\frac{d_1}{d_2}\right)^{0.41}\left(\frac{n_2}{n_1}\right)^{0.41}\left(\frac{C_{C2}}{C_{C1}}\right)^{0.41}. \quad \text{(S1)}$$

Using Eq.(3), the ratio between C_{C2} and C_{C1} becomes

$$\frac{C_{C2}}{C_{C1}} = \frac{d_2 d_1 + 3 d_1 \lambda}{d_2 d_1 + 3 d_2 \lambda} \quad . \quad \text{(S2)}$$

Using Eq.(5) and the following Gauss-Legendre quadrature rule,

$$\ln(1+x) = \frac{6x+3x^2}{6+6x+x^2} \quad , \quad \text{(S3)}$$

the ratio between n_1 and n_2 is expressed as follows,

$$\frac{n_2}{n_1} = \frac{\mathrm{d}_2}{\mathrm{d}_1} \quad . \quad \text{(S4)}$$

By inserting Eq.(S2) and Eq.(S4) into Eq.(S1), the ratio between W_{p1} and W_{p2} becomes as follows,

$$\frac{W_{p1}}{W_{p2}} = \left(\frac{d_2 d_1 + 3 d_1 \lambda}{d_2 d_1 + 3 d_2 \lambda}\right)^{0.41} \quad . \quad \text{(S5)}$$

If we put the diameter of smaller particles (virus, antibody) to $\mathbf{d_1}$ and the diameter of larger particles (bound virus-antibody) to $\mathbf{d_2}$, and also put the charge number of smaller particles (virus, antibody) to n_1 and the charge number of larger particles (virus, antibody) to n_2, we can easily find that the ratio between W_{p1} and W_{p2} is smaller than 1.

Current decrease due to the deposition of virus–antibody bound particle

The reduced current in the swCNT-FET that was observed in the presence of virus–antibody bound particle was most likely due to the donation of electrons from negatively charged virus–antibody bound particles. Because of the *p*-type characteristics of the swCNT, the donation of electrons would reduce the number of holes (*p*-type charge carriers) in the swCNT, resulting in a decrease in the source–drain current. Details are explained with the following figure.

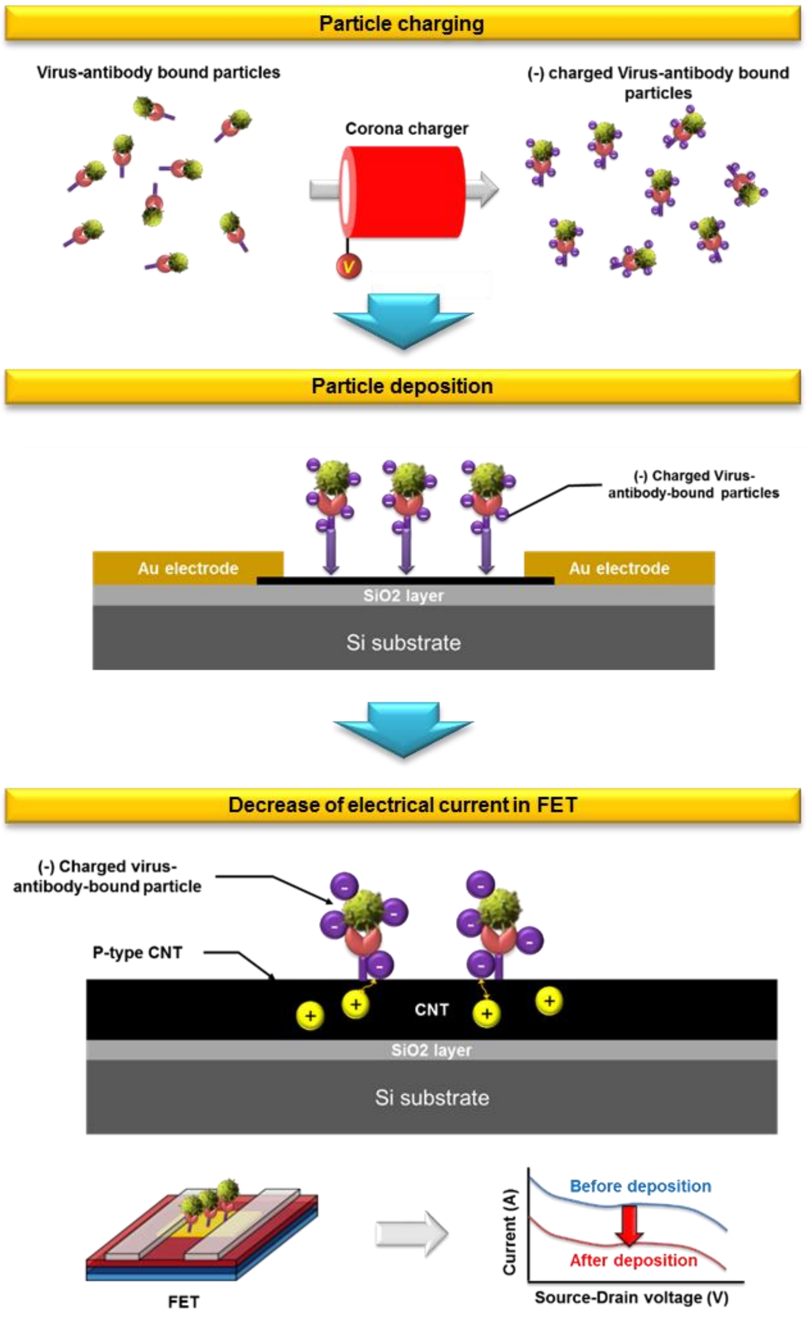

Figure S2. Effect of charged virus-antibody bound particles on *I-V* characteristics of swCNT-FET (K. –T. Park drew the figure by using Microsoft PowerPoint).

Detection of aerosolized bacteriophage MS2 virus using a CNT FET

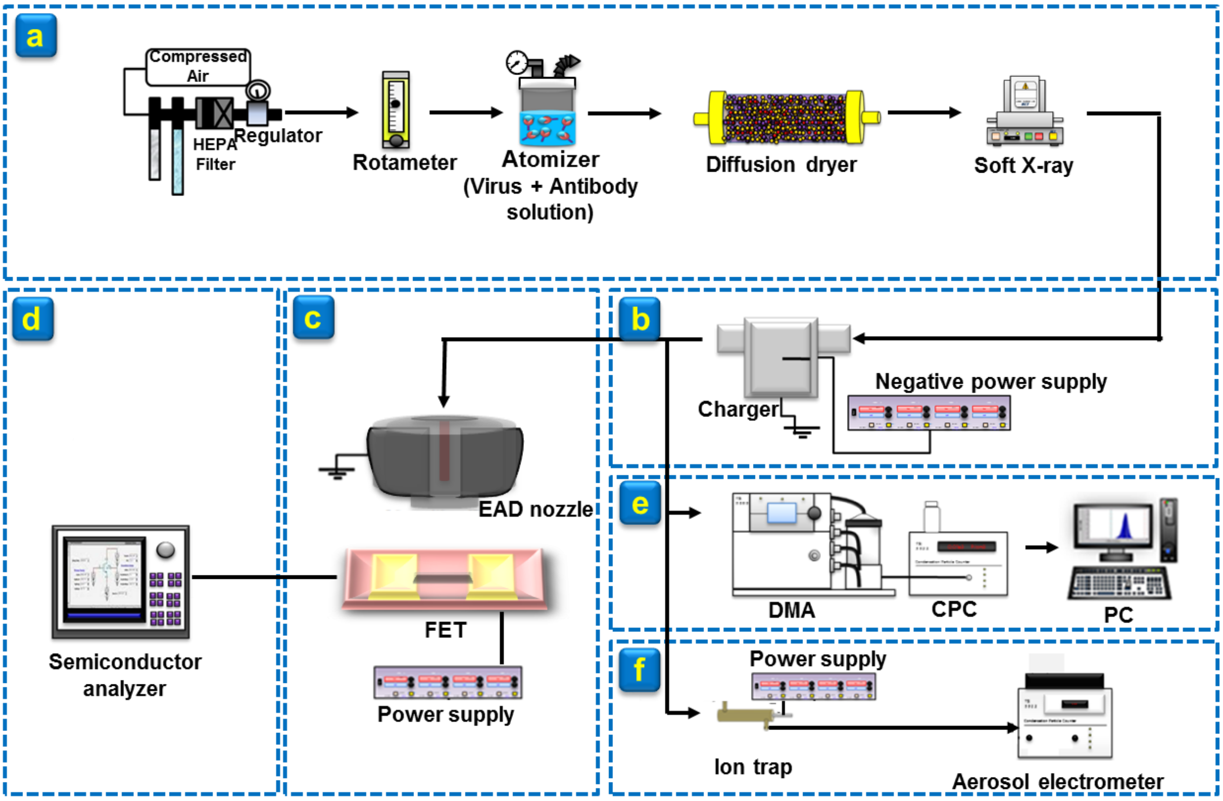

Figure S3. A schematic diagram of the experimental setup (K. –T. Park drew the figure by using Microsoft PowerPoint). (a) Virus–antibody binding and aerosolization, (b) particle charging, (c) electro-aerodynamic deposition, (d) analysis of the characteristics of the FET, (e) measurement of particle size distribution, and (f) measurement of particle charge number.
